# AGD: Aneurysm Gene Database

**DOI:** 10.1093/database/bay100

**Published:** 2018-09-26

**Authors:** Ruya Sun, Chunmei Cui, Yuan Zhou, Qinghua Cui

**Affiliations:** 1 Department of Biomedical Informatics, Department of Physiology and Pathophysiology, Center for Noncoding RNA Medicine, MOE Key Lab of Cardiovascular Sciences, School of Basic Medical Sciences, Peking University, Beijing, China; 2 Center of Bioinformatics, Key Laboratory for Neuro-Information of Ministry of Education, School of Life Science and Technology, University of Electronic Science and Technology of China, Chengdu, China

## Abstract

An aneurysm is an outward bulge on an arterial wall. Aneurysms are becoming a serious public health concern as the worldwide population ages. Unfortunately, no effective drugs have been developed for aneurysms to date. In addition, aneurysms may be associated with grave prognosis due to conditions such as ruptures and recurrence. Altogether, these factors make earlier aneurysm prevention, diagnosis and intervention strategies even more important. A bioinformatics resource for aneurysm-associated molecules would be helpful for addressing the above issues; however, such a tool is not yet available. In this study, we developed Aneurysm Gene Database (AGD) for the above purpose. AGD contains 1472 aneurysm-gene associations, including 29 types of aneurysms, 967 protein-coding genes, 29 miRNAs, 6 lncRNAs and several other types of molecules. Users can search, browse and download content in AGD. We believe that AGD is a valuable resource that can help us better understand aneurysms and discover novel treatment targets.

## Introduction

An aneurysm is defined as an outward bulge on an arterial wall and is associated with significant morbidity and mortality (1, 2). As the worldwide population ages, aneurysms have become a major public health concern. Since aneurysms may occur anywhere along the full length of the aorta (3), they can be classified into several types based on their location, such as thoracic aortic aneurysm, abdominal aortic aneurysm (AAA) and carotid aneurysm.

The natural course of an aneurysm is asymptomatic growth followed by abnormal vasodilatation, arterial occlusion, vasospasm, aortic dissection and, at worst, rupture (4–7). For example, previous studies have shown that the prevalence of AAA is 4–8% among men >65 years (8–12). For people aged >45 years, the out-of-hospital death rate reaches up to 80% for AAA rupture. Aneurysm rupture is significantly lethal, and while acute mortality is lower, it still reaches 50% even when patients ultimately undergo surgery (13–15).

Unfortunately, although aneurysm prognosis can be devastating, the pathological mechanisms contributing to aneurysm are still poorly understood (16). Currently, no viable drugs are available for aneurysm, and conventional treatment options are limited to surgical or endovascular intervention (17, 18). Therefore, early aneurysm prevention, diagnosis and intervention are of vital importance, especially for unruptured aneurysms. A web-based resource for aneurysm-associated genes will be helpful to not only better understand the mechanism of aneurysm formation and development but also discover preventive and therapeutic targets and drugs; however, such a tool is not yet available.

Rapidly changing high-throughput technologies offer an opportunity to change the above situation. High-throughput technology, which is characterized by big data production ability, provides clinically relevant information (19, 20). We can identify potential genes associated with diseases by integrating information at different omics levels (21). On one hand, these associations enable the definition of candidate biomarkers for early prevention, diagnosis and disease monitoring (22). On the other hand, the associations may help researchers obtain a better understanding of disease and explore underlying pathological mechanisms, which can indicate actionable therapeutic targets.

**Figure 1:**
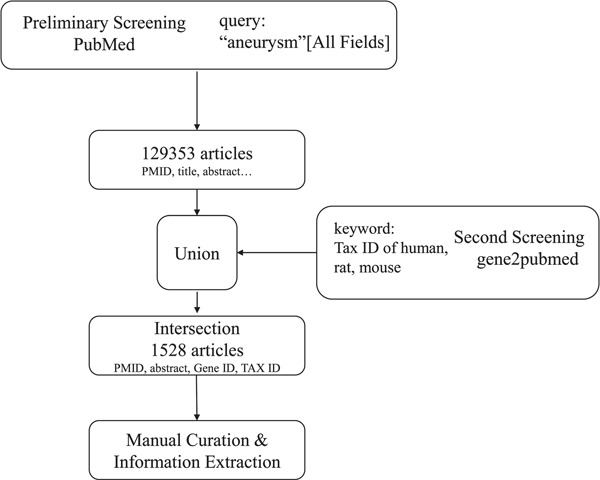
Workflow of literature mining and manual curation.

Here, we constructed AGD (Aneurysm Gene Database), a database of aneurysm-gene associations. AGD integrates information obtained from PubMed and 5 other research sources, and to date, it contains 1472 curated aneurysm-gene pairs, including 967 protein-coding genes, 29 miRNAs, 6 lncRNAs and 29 aneurysm types from 3 species (human, mouse and rat). As an online, open-access user-friendly database, AGD provides an interface with search, browse and download functions. We believe that AGD can help clinical and biological researchers explore molecular mechanisms and novel actionable therapeutic targets related to aneurysms.

## Materials and methods

### Database construction

Three steps were taken to initially construct the database: (i) literature mining and manual curation of PubMed abstracts, (ii) supplementing the database with information obtained from five other scientific resources and (iii) integrating all the information in a uniform format.

#### Step 1: literature mining and manual curation

As the workflow shows in Figure 1, the collection of aneurysm-gene associations began with literature mining on PubMed (www.ncbi.nlm.nih.gov/pubmed). In the preliminary screen, we searched PubMed using the query (‘aneurysm’ [All Fields]), and it returned 129 353 articles and relevant information [PubMed Identifier (PMID), title, author, abstract etc.]. Since most of the 129 353 articles containing ‘aneurysm’ were not related to aneurysm-gene relationships and human, mouse and rat were the only species we were interested in, a secondary screening was performed. Then, we obtained information from the Entrez Gene2PubMed file of the National Center for Biotechnology Information (NCBI, http://www.ncbi.nih.gov/gene), which records relationships between genes and publications and provides corresponding Gene IDs, PMIDs and Tax IDs. Next, entries matching the condition that Tax ID = 9606 (*Homo sapiens*), 10116 (*Rattus norvegicus*) or 10090 (*Mus musculus*) were selected. We obtained the primary result by taking the intersection of the above results, which included the PMIDs, abstracts, Tax IDs and Gene IDs of 1528 articles containing ‘aneurysm’ that were focused on genes.

The next step was to extract information from the 1528 articles. This step was manually curated to ensure that the association between genes and aneurysms existed and were significant and that sufficient details were recorded for the associations. The curation criteria were as follows: (i) aneurysm is at least the main symptom described in the article, (ii) aneurysm-gene associations existed and were significant (*P* < 0.05 for experiment results and epidemiological findings) and (iii) case reports were not excluded but had to be noted.

#### Step 2: aneurysm-gene associations from other resources

Some aneurysm-gene associations may not be included in the above results for many reasons. We mined additional aneurysm-gene associations by collecting information from five other scientific resources: GWASdb v2 (http://jjwanglab.org/gwasdb) (23), Online Mendelian Inheritance in Man (OMIM) (http://www.omim.org/) (24), Gene Expression Omnibus (GEO) (https://www.ncbi.nlm.nih.gov/geo/) (25), Human Genome Mutation Database (HGMD) (http://www.hgmd.cf.ac.uk/ac/index.php) (26) and ClinVar (http://www.ncbi.nlm.nih.gov/clinvar/) (27). For GEO, Wilcoxon test was used to confirm the associations between genes and aneurysms, and only results that met the condition of False discovery rate (FDR) < 0.05 were included. For OMIM, GWASdb v2, HGMD and ClinVar, we searched each database using the query ‘aneurysm’ and extracted detailed information from the search results.

#### Step 3: integration

To integrate all the information obtained in steps 1 and 2, we first needed to ensure that information from different data sources was compiled in a single format. This detailed information was composed of nine parts: (i) gene ID, (ii) official gene symbol, (iii) type of aneurysm (if aneurysm was just the main symptom of a disease, the disease name was recorded instead; if the specific type of aneurysm was not given, it was recorded as ‘Aneurysm’), (iv) description [of the association between the gene and aneurysm, including all details, such as mutation pattern, changes in the patient group (in DNA, protein or RNA level), and histological origin of samples], (v) data source (PMID, GSE, MIM or ClinVar number from the data source, which includes evidence supporting the gene-aneurysm association), (vi) more info (such as a description of the surveyed population, aneurysm characteristics and accompanying symptoms), (vii) clinical information (such as whether or not the gene can be used as a biomarker for aneurysm diagnosis, monitoring and prognosis and whether the gene has any protective potential), (viii) subcellular location (of gene products, helping users get deeper understanding of associated genes) and (ix) species (human, mouse or rat).

### Database design

AGD is built in Django 1.5.5, and the data are managed by SQLite3. As a freely accessible database, users can visit AGD via all common browsers, such as Chrome, Internet Explorer, Safari and Firefox. For user convenience, AGD offers an interface with browse, search, download and submit functions, and researchers can obtain all AGD entries in text or Excel format.

## Results

### Database content

Currently, a total of 1472 aneurysm-gene associations across 3 species (human, mouse and rat) are documented in AGD, including 29 types of aneurysms, 967 protein-coding genes, 29 miRNAs, 6 lncRNAs and several other types of molecules. For each aneurysm-gene pair, relevant details are provided: official symbol, gene ID, aneurysm type, description, data source, more information, clinical information, subcellular location and species. In terms of ‘description’, the most important aspect, data not only at the DNA level but also at the protein and RNA levels are recorded. As for ‘aneurysm type’, diseases, such as Marfan syndrome, are also included because aneurysm represents their main complication or symptom.

### Web interface

**Figure 2:**
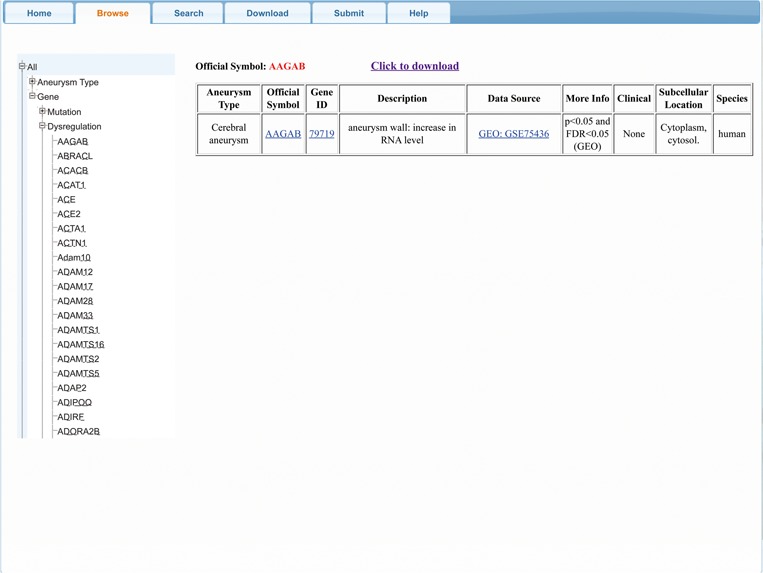
AGD interface.

AGD provides a user-friendly interface with four main functional regions (Figure 2), allowing users to browse, search and download AGD entries and submit new entries to enrich the database.

### Browse

The ‘Browse’ function provides a comprehensive overview of aneurysm-gene pairs associated with a specific gene or aneurysm type. After clicking on ‘Browse’ on the control bar, users must first choose the species of interest (we also set an option named ‘all’ to select all three species). Next, the user must choose between ‘gene’ and ‘aneurysm type’, depending on their interest. Under ‘gene’ option, two (or three) categories are provided: ‘Mutation’, ‘Dysregulation’ [and ‘Others’ (means genes involving neither mutation nor dysregulation)]. Once these selections are made, a certain subset of genes or aneurysm types will be selected and shown on the webpage. When the user selects one entry from the subset, the relevant result will then be shown. For users’ convenience, ‘gene ID’, ‘official symbol’ and ‘data source’ are listed in the results table and are linked to their corresponding scientific resources (gene for gene ID and official symbol, PMID, MIM, ClinVar and GEO number for data source or source file) to obtain further information.

The ‘Browse’ results can be accessed by clicking the ‘Click to download’ button at the top of the results table; users can obtain these results in a text file.

### Search

The ‘Search’ function allows user to search for a specific gene of interest. Here, we set four keywords for the query. The search query starts with two pull-down display menus, one for species and the other for aneurysm type. Options are given as a default for these two menus; users just need to pick one from the list. If the user is unsure which option to select, we also provided an option of ‘all’. The last step is typing in the official symbol of the gene(s) of interest. However, before this step, there is a check box set to search mode. Users who select the ‘exact’ search mode must input the precise gene symbol in their search (case insensitive) and they could search for several genes at a time. Otherwise, if the users select the ‘fuzzy’ search mode, they can just input part of the official symbol (case insensitive). Regardless of the search mode, if the user is unsure about which gene to search for or if he/she does not type in anything for the last keyword (‘Gene Symbol’), the default value (‘all genes’) is applied.

After obtaining all four keywords, the search results are provided. Users only need to click the ‘Reset Search’ button to start a new search.

### Download

Since AGD is an open-access, user-friendly database, all AGD entries can be obtained with the ‘Download’ function to help users with their studies. Depending on the selected species, we offer four data sets: separate entries for human, mouse and rat and all three species combined. For each data set, two formats (Excel and text) are provided for the download file.

### Submission

Currently, AGD collects relevant information from only five scientific resources and PubMed abstracts before March 2018. However, with the emergence of more studies on aneurysm-gene associations, the number of related articles increased almost every day, and new candidate aneurysm-gene associations are constantly discovered. The content of AGD is by no means real-time or complete, and some genes associated with aneurysms are left out, which makes it necessary to regularly update AGD. A submit function is provided to make AGD as complete as possible. This function allows researchers to submit newly discovered aneurysm-gene associations and their reference information. After curating and confirming the submitted information, we will add it into the database.

### Future extensions

Considering that AGD was developed to allow researchers to trace the trend of aneurysm research in a less time- and effort-consuming way, regular updates seem to be necessary. As mentioned previously, the data are managed by SQLite3, and the addition of new entries is quite simple. For now, the plan is to update AGD annually.

In addition, in recent years, researchers have become increasingly interested in non-coding RNA (ncRNA). Researchers believe that ncRNAs, such as long nRNAs (lncRNAs) and microRNAs, were previously underestimated and are very important pieces in the puzzle of life science (28–30). A small number of microRNAs and lncRNAs (e.g. MIR195 and LINC00982) have been collected in AGD, and we may add more ncRNA-associated entries in AGD in the future.

### Conclusion

Aneurysm is an irreversible and permanent cardiovascular disease with high incidence and poor prognosis. To date, no viable medications for aneurysms are available. Therefore, the most realistic current challenge is to uncover the underlying molecular mechanisms of aneurysm and discover actionable therapeutic targets. Biomarkers of aneurysm are also crucial for making diagnosis, monitoring disease progress, establishing treatment plans and predicting prognosis.

We developed AGD, a comprehensive database of aneurysm-gene associations, to solve these problems. With the help of the AGD data set, researchers may discover mechanisms contributing to aneurysm by analysing the data set and associated signaling pathways and conducting functional enrichment analysis, etc. AGD contains 1472 gene-aneurysm pairs across 3 species, including 29 types of aneurysms, 967 protein-coding genes, 29 miRNAs, 6 lncRNAs and several other types of molecules.

We believe that the information recorded in AGD will be helpful for basic research and clinical applications; we may eventually shed light on aneurysm pathogenesis and be able to treat it.

## Notes

**Database URL**: http://www.cuilab.cn/agd
